# Three-Dimensional Technology Applications in Maxillofacial Reconstructive Surgery: Current Surgical Implications

**DOI:** 10.3390/nano10122523

**Published:** 2020-12-16

**Authors:** Yasmin Ghantous, Aysar Nashef, Aladdin Mohanna, Imad Abu-El-naaj

**Affiliations:** 1 Department of Oral and Maxillofacial Surgery, Baruch Padeh Medical Center Poriya, Tiberias 158001, Israel; yasmen.ghantous@gmail.com (Y.G.); ma.aladdin@gmail.com (A.M.); iabu@poria.health.gov.il (I.A.-E.-n.); 2 The Azrieli Faculty of Medicine, Bar Illan University, 8 Henrietta Szold St, Safed 1310000, Israel

**Keywords:** three dimensional printing, 3D printing, oral and maxillofacial reconstruction, 3D printing in the cranio-maxillofacial surgery

## Abstract

Defects in the oral and maxillofacial (OMF) complex may lead to functional and esthetic impairment, aspiration, speech difficulty, and reduced quality of life. Reconstruction of such defects is considered one of the most challenging procedures in head and neck surgery. Transfer of different auto-grafts is still considered as the “gold standard” of regenerative and reconstructive procedures for OMF defects. However, harvesting of these grafts can lead to many complications including donor-site morbidity, extending of surgical time, incomplete healing of the donor site and others. Three-dimensional (3D) printing technology is an innovative technique that allows the fabrication of personalized implants and scaffolds that fit the precise anatomy of an individual’s defect and, therefore, has attracted significant attention during the last few decades, especially among head and neck surgeons. Here we discuss the most relevant applications of the 3D printing technology in the oral and maxillofacial surgery field. We further show different clinical examples of patients who were treated at our institute using the 3D technology and discuss the indications, different technologies, complications, and their clinical outcomes. We demonstrate that 3D technology may provide a powerful tool used for reconstruction of various OMF defects, enabling optimal clinical results in the suitable cases.

## 1. Introduction

The repair of large oral and maxillofacial (OMF) defects, secondary to tumor, trauma, or congenital disease, employs a multidisciplinary approach and represents one of the most difficult and challenging areas in head and neck surgery. The goals of craniofacial reconstruction include, mainly, the restoration of complex functional, anatomic, and aesthetic characteristics, with important respect to the craniofacial growth in the growing patients. To this end, autologous bone grafts remain the gold standard in hard-tissue reconstructive surgery owing to their osteoinductive and osteoconductive properties, osteogenic properties and the potential for continuous growth of particular autologous grafts at the defect sites (i.e., costochondral graft) [1,2]. Moreover, in defects with extensive hard and soft tissue loss in the OMF complex, loco-regional flaps and microvascular free tissue transfer is still considered as the superior reconstructive option [3,4]. However, despite high success rates of both vascularized and non-vascularized grafts, such reconstructive options still have critical disadvantages including, mainly, donor-site morbidity, availability in limited quantities, prolonged anesthesia time, unpredictability of bone graft resorption, total flap loss, and the need to manually sculpt the graft into the shape of the defect site [4,5,6].

The use of biomaterials for bone regeneration in large OMF defects is promising, however, those materials must meet specific characteristics in order to regenerate new and functional bone; for example, biocompatibility, porosity, morphology and inter-connectivity, osteoconductivity/osteoinductivity, biodegradability and several specific mechanical characteristic that enable suitable handling and growing. Unfortunately, there are few biomaterials that fit those requirements, especially for large defects.

Three-dimensional (3D) printing is a novel technique that has evolved over the past three decades and has the potential to revolutionize the field of reconstructive medicine in general [7,8]. Since its first description by Hideo Kodama in 1981 [9], 3D technology has matured and many more sophisticated different printers than the original machines currently exist, allowing for application in a range of fields including aerospace, engineering, consumer products, arts, food industry, education, manufacturing, and medicine [8,10]. Three-dimensional printing is also defined as additive manufacturing (AM), and this technique uses metals, ceramics, and plastic material to produce three-dimensional (3D) objects for the usage in different disciplines, including medical application [11]. The AM process is defined by the International Organization for Standardization (ISO) and American Society for Testing and Materials (ASTM) as the “process of joining materials to make parts from 3D model data, usually layer upon layer, as opposed to subtractive and formative manufacturing methodologies”. [12]. The processes encompassed in AM are the 3D analog of the very common 2D digital printers; therefore, AM is also commonly referred to as 3D printing. AM has gained to many definitions over the last 30 years, such as direct digital manufacturing, additive layer manufacturing, additive fabrication, additive processes, free-formed fabrication, solid free-formed fabrication, rapid manufacturing, and rapid prototyping [13]. It is noteworthy that, in contrast to the conventional manufacturing processes (i.e., subtractive and formative manufacturing processes), AM technology has the ability to deal and create complex geometric products [14], with a high degree of functionality [13] and low cost of manufacturing [15]. Thus, AM is considered as the ideal technology for producing unique 3D objects that are manufactured in low volumes that are generally used for medical and dental applications [16,17,18].

In this review, we discuss the three principal applications of AM process that are relevant to oral and maxillofacial surgery including: (i) the use of 3D printing to generate 3D models for surgical planning and education; (ii) the use of 3D printing technology for the production of patient-specific implants (PSI); and (iii) the bio printing of organic structures. We provide different clinical cases where AM process is applied for treatment planning, surgical stimulation, intraoperative guidance and printing of PSIs for reconstruction of OMF defects. We also provide an overview of the printing technologies that are most commonly used for oral and maxillofacial surgery applications.

## 2. Three-Dimensional (3D) Printing Techniques

In the medical field, and particularly, in the oral and the maxillofacial reconstructive surgery, there are several variants of AM processes and printers available today [8,10,12,19]. However, all AM process share the same concept of work-flow which can be summarized as follows [11,15]: the process begins with capturing anatomical scans using imaging techniques such as magnetic resonance imaging (MRI) and computed tomography (CT) scans; then, a computer aided design (CAD) model is processed and optimized using specific computer techniques. Then, the CAD model is transformed into a standard triangulation or tessellation language (STL) file and imported into an AM setup. Each AM model, is formatted in the STL to a geometric shape, and sliced into thin layers and the movement of the depositing or fusing unit (“printing head”), and substrate (“printing platform”), as well as other parameters are programmed by specialized software. Consequently, the AM machine constructs the 3D model layer-by-layer according to a specific and precise programmed parameters., the built object is removed from the building platform and followed by post-processing procedures (such as polishing, coating, or thermal treatment) to obtain a functional part.

### 2.1. Stereolithography

In stereolithography (SLA), the 3D model is fabricated in a series of layers that correspond to the axial image slices of the CT scan. The technology is classified as a vat photopolymerisation AM process in which an ultraviolet (UV) light is projected on a bath of curable photopolymeriser resin. After the first layer is built, it either moves, gradually, out of the bath or descends depending on the production configuration, and the focused energy beam renders the next layer, according. Typically, each layer is polymerized at a thickness of 0.05–0.15 mm. This process is continued until each corresponding slice of the CT image is duplicated in the resin model. In medical field, and in particular in OMF surgery, the generated SLA models are, mostly, prepared by acrylate or epoxy resin, and used for surgical guides and templates, as well as for training residents, designing soft tissue incisions, surgical resection margins, assessing of bony defects for grafting, adaptation and pre-bending of reconstruction plates, and fabrication of custom prostheses. The accuracy of these printed objects in resembling the human anatomy as well as its utility in the perioperative management for improving the predictability of treatment of maxillofacial defects secondary to traumatic or pathologic conditions have been confirmed in numerous reports [20,21,22,23,24,25,26,27].

### 2.2. Laser Sintering

Laser sintering (LS) and related techniques (i.e., selective laser sintering, direct metal laser sintering, laser melting and others) are classified as a powder bed fusion process of AM that is currently employed, widely, in medical disciplines. The process is based on the same principle of layer-by-layer AM. The system normally consists of a laser, an automatic powder layering apparatus, a computer system for process control and some accessorial mechanisms such as gas protection systems and powder bed preheating systems. The function of a LS system employs a focusing of a high-powered energy laser into a powdered substrate, causing a fusion of the substrate into the desired shape. Once a layer of substrate has been sintered, a new layer of substrate is added on the top of the developing construct, and energy is applied again [28]. Different types of laser are used for this purpose (including CO_2_, Nd:YAG, fiber lasers, disc lasers and others) and selected based on to the laser absorptivity of the specific material used and the operative metallurgical mechanism of the powder densification [29,30]. The process is include firstly a leveling and fixation of the substrate on the building platform, followed by deposition of a thin layer of loose powder (normally ~100 µm) on the substrate. Subsequently, a laser beam scans the powder bed surface to form a layer according to the CAD data. The procedure is repeated, in a layer-by-layer manner, until a complete highly accurate and nearly a full density functional part is produced [28]. This technology has traditionally been used in non-biological printing, but also for biological substrates [8]. Indeed, the LS technologies have changed the workflow for various surgical procedures among many disciplines within the OMF surgery field during the last years. The availability of this process provided us with the ability to fabricate a wide range of objects including surgical osteotomy guides with high accuracy, custom-made titanium orbital floors, custom made grids, sub-periosteal dental implants, custom-made cranial plates and other parts that perfectly adapt to the specific anatomical requirements of patients [31,32,33,34,35,36,37,38,39].

### 2.3. Extrusion Printing

Extrusion printing is another widely available process for 3D printing of biological and non-biological materials and considered among the most widely used AM processes, especially when dealing with polymers and thermoplastic composites. This process includes, mainly, the fused deposition modeling (FDM) technique and the fused filament fabrication (FFF). The basic principle of material extrusion additive technology involves the loading and liquefaction of a printed material. The material moves through a nozzle or orifice by applying a pneumatic pressure, followed by plotting of the liquefied material according to a pre-defined path in a controlled manner, and layer-by-layer bonding of the material to itself or a secondary build material to form a coherent solid structure. Once a layer is formed, the build platform moves down or the extrusion head moves up, and a new layer of material is deposited and adhered onto the previous layer. In contrast to other AM techniques, the extrusion printing process allow for multi-material deposition due to the possibility of adding one or more extrusion unit simultaneously and can be used for various thermoplastics for the same product [40,41]. Depending on the type of extruder used, one can classify material extrusion additive manufacturing into main three different types; plunger-based, filament-based, and screw-based [42]. Indeed, material extrusion of filaments was first patented by the company Stratasys and commercialized as fused deposition modeling (FDM) [43]. This process of AM is popular in the medical field due to its safe and simple fabrication process because of no powders, lasers, solvents, nor volatile compounds are used, the low cost of the equipment, and the availability of a great variety of filaments for printing. During the last few years, the use of FDM technology for OMF reconstructive surgery was restricted mainly to manufacturing surgical guides for preoperative planning of complex surgical treatments. However, recently the technology was successfully used to print alloplastic materials, named polyetheretherketone (PEEK), which has emerged as an attractive option for producing PSI owing to its excellent combination of high-temperature performance, chemical resistance, fatigue resistance, lightweight, high yield strength, stiffness, and durability [44,45].

## 3. Three-Dimensional Printing Materials

The fabrication process of each 3D printing includes external heat, light, laser and other energy sources. The mechanical characteristics of the different materials and the variable chemistry enable it to react optimistically to the different external source of energy and to transform to the desired shape. Nowadays, the advanced 3D printing technologies enable shape transforming of the materials, layer by layer, in response to the external energy source. There are several material states available, such as powder, pellets, resin, and granules, while the specific material type and characteristics are developed in accordance with the expanded development of 3D manufacturing.

The most popular AM materials are plastic nylon, and polyamide, since both are strong and flexible, and basically white in color. They can be used in two forms, powder and filament. Powder is used mainly in the sintering process, and filament is mainly used in FDM [46]. If a different range of color is desired, Acrylonitrile butadiene styrene (ABS) could present a suitable choice, ABS is a strong, filament plastic material and it is available in a wide range of colors. Polylactic acid (PLA) is also a plastic material available both in filament and resin forms, in addition it is available in several colors, this material can be used for the FDM process, where in resin form it can be used for digital light processing, the main drawback of this material being its rigidity, and non-malleability. Alumide, is a powder format plastic material that is used for sintering, this material is formed by combining Polyamide in its powder format with powdered aluminum. Ceramics are relatively a new group of 3D materials that have proved to be suitable for several medical applications, however, the printed ceramic objects should undergo post-processing firing and glazing to achieve a smooth surface area [46,47]. Another popular group of materials are metals, while the most common metal composites used are aluminum, titanium, and cobalt derivatives. Stainless steel is one the metal materials most often used in 3D printing due to its strength, it is naturally silver, but it can be blended with other materials to gain a variety of other properties. Research is being undertaken to evaluate the use of bio materials for 3D printing for medical applications.

Simple direct media layer (SDL) process-based printers provide a professional 3D printing technique, and this technique enables the use paper-based 3D printers; such materials have many advantages, they are safe, easily recycled and require no post processing [47].

## 4. Clinical Examples of Additive Manufacturing (AM) Use in Oral and Maxillofacial Surgery

The following section of the paper is focusing on clinical case reports that were treated at our department with emphasis on the indications for use, material of choice, intra-operative and post-operative complications. The demographic characteristics, treatment indications, and clinical outcome of patients who were treated with the 3D application at our institute between 2015–2020 were reviewed, retrospectively. The institutional review board of Peda-Poria hospital approved the study protocol. Briefly, computed tomography (CT) scans were obtained for these patients. Images from these modalities were saved in a digital imaging and communications in medicine (DICOM) format. Subsequently, CAD software were used to create a virtual 3D prototype, based on the surgery plan. Standard tessellation Language (STL) format was then generated to allow 3D printing and deposition of the material layer by layer to achieve the final 3D object. Depending on the application, an appropriate printing technique and printer was selected (i.e., SLA, SLS etc...). Finally, final post printing modification of the printed part was performed [8,10,12,19].

A total of 16 patients were treated at our department between 2015 and 2020, using the AM process, and are summarized in Table 1. The mean age of patients was 45.5 years (range 19–80 years). The male to female ratio was 8:8. The technology was mostly applied for the trauma and post-trauma surgery discipline (7/16; 44% of cases), followed by pre-prosthetics surgery discipline (5 out of 16, 31%), oncologic surgery discipline (2 out of 16; ~13%), one case of temporomandibular joint (TMJ) surgery and one case for facial deformity correction surgery. PSI was the most printed object (10 out of 16) in our case series and were used mainly for floor of orbit reconstruction (4 out of 10 cases; 40%). Titanium material was the most used material in 3D printing with 69% of cases (11 out of 16 cases). PEEK material was used in three cases for PSI printing as a reconstruction approach of the floor of the orbit, nasal bone and temporal and frontal bone reconstruction. Intraoperative complications were noted among three cases (19%- two PSI and one surgical cutting guide) and were to include mainly loss of accurate fitting of the printed object; in these cases, minimal adjustment of the printed part was performed intra-operatively allowing for acceptable fitting. With regard to post-operative complications, one case has showed extensive post-operative edema followed by exposure of PSI and development of acute infection, this patient was retreated successfully with a free flap fibula reconstruction. In one patient, who was treated for nasal bone reconstruction, this showed an insufficient esthetic of nasal contour. As expected, almost all of the cases with PSI showed some degree of postoperative edema.

## 5. AM Process in Virtual Surgical Treatment Planning, Surgical Stimulation and Education

**Figure 1:**
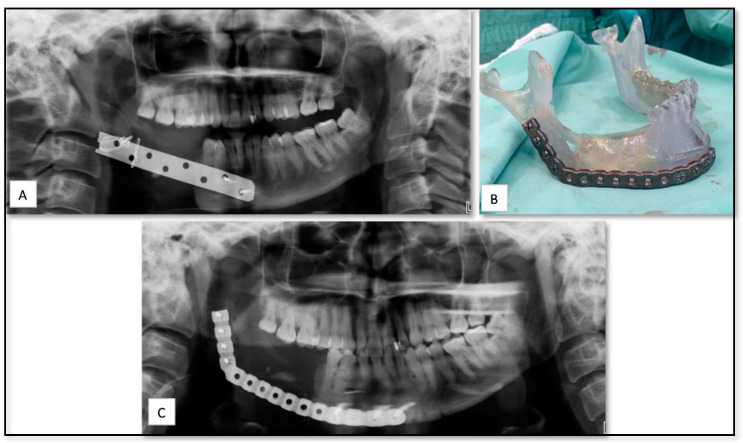
(**A**) Panoramic radiograph showing right mandibular defect with the old bone plate. (**B**) Stereolithographic (SLA) model and the prebent reconstruction plate. (**C**) Post-operative panoramic radiograph showing the installed reconstruction plate.

SLA is a valuable adjunct to traditional methods of treatment planning and surgical stimulation for reconstruction following resection of tumors, developmental abnormalities, or trauma reconstruction. In practice, SLA aids in patient education, clarification of diagnoses, and improving treatment planning. These models allow case-specific surgical simulation and are used as a template for modification of bone plates or the fabrication of implants, which may improve the workup and operative phases and can enhance the surgical treatment [20]. Here, we present a 33-year-old woman who was referred to our institution for evaluation and treatment plan 8 years after a gunshot wound injury (GSW) to her right mandible (Figure 1). She was treated by other surgeons with bone plates 8 years ago, but infection developed at the surgery site and a fistula was noted. Her first management included the removal of the infected plate and a wound closure. In addition, because of a large defect in her mandible body, the patient elected to undergo reconstruction using bone plates with subsequent bone graft. A SLA model was constructed to pre-bend the bone plates in order to re-create this patient’s pre-injury bony contour and allow for adequate mandible strength. Prior to surgery, a mandibular reconstruction plate was prebent using the printed SLA model as a reference and screw placement was also planned, as well as screw lengths, which were recorded by measuring the thickness of the model at each plate hole. The final post-operative result showed adequate reconstruction of facial contours and adequate facial symmetry.

### 5.1. AM for Manufacturing of Surgical Guides for Zygomatic Implants Insertion

**Figure 2:**
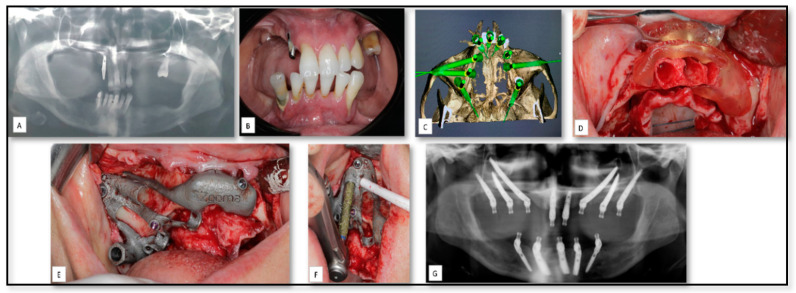
(**A**,**B**) Pre-operative panoramic and clinical view of partially edentulous atrophic posterior maxilla and maxilla. (**C**) Implants planned based on the prosthetic needs. (**D**) Cutting guide for alveoloplasty before implants placement. (**E**) The installation of the created surgical guide. (**F**) Implant osteotomy guided by the surgical guide. (**G**) Post-operative panoramic view showed the implants opposition as planed preoperatively.

One of the most printed 3D objects in the OMF surgery are surgical guides that are designed to facilitate the orientation and execution of drillings, permitting a correct dental implant placement and angulation, as predicted in preoperative planning [48,49,50]. Here, we show a 56-year-old female patient who was referred to our clinic because of severely atrophic posterior mandible and maxilla (Figure 2). The treatment plan included placing two conventional, four zygomatic and two pterygoid implants with immediate loading principle. Mandibular prosthesis was planned with five implant supported fixed partial denture. Indeed, zygomatic and pterygoid implant implants have become a predictable treatment modality for the rehabilitation of the severely atrophic maxilla [49]. However, due to different anatomic variations, proximity to vital anatomic structures and limited intraoperative visibility, the placement of such implants can be a challenging procedure and may ultimately lead to postoperative surgical and prosthetic complications [51]. A prosthetically driven preoperative planning was performed and a 3D metal drill guide was fabricated and used to allow full control of the accurate location and angulation of the implants.

### 5.2. AM for of Pre-Prosthetics Patient-Specific Implant (PSI) Manufacturing

**Figure 3:**
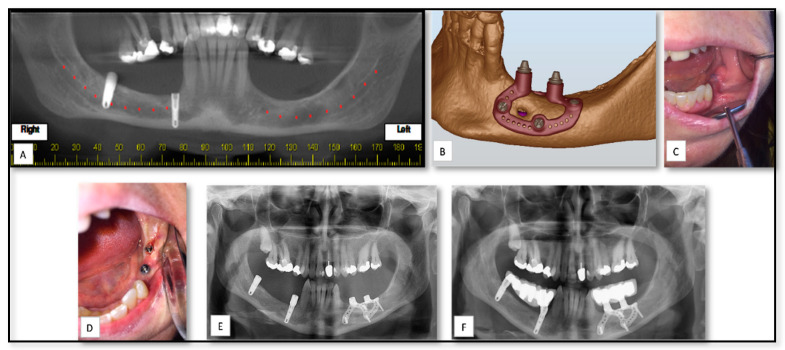
(**A**) Preoperative Cone beam computed tomography (CBCT) panoramic showed posterior edentulous mandible. (**B**) Pre-surgical planning and modeling of the sub-periosteal implant (**C**) One week after placement of the sub-periosteal implant shows proper healing. (**D**) One week after implant coverage and installation of dental healing caps. (**E**,**F**) Postoperative panoramic view showed the sub-periosteal implant with and without the final dental rehabilitation.

Endosseous dental implants provide a highly predictable solution for the prosthetic rehabilitation of partially and totally edentulous patients, with high rates of survival and success in the medium and long terms. Insertion of such implants requires the existence of adequate quantity (volume) and quality (density) of bone at the surgical site [50]. Several surgical technique have been proposed to restore bone volume to a level that allows the proper implant placement in cases of patients with severe bone atrophy, including inlay/inlay bone grating [52,53], guided bone regeneration (GBR) with resorbable or non-resorbable membranes [54,55], alveolar ridge split, distraction osteogenesis [56,57], and maxillary sinus augmentation. However, theses surgical techniques are complex and can have a rather high percentage of complications. The new direct metal laser sintering techniques available today provide the ability to fabricate custom-made implants [56]. Briefly, a subperiosteal implant is a type of dental implant that is placed between the periosteum and the residual alveolar bone [58]. It usually has two to four trans-mucosal elements projecting through the mucosa into the oral cavity, connecting the implant to the prosthesis. Here, we show an example of a patient with left posterior severe atrophic mandible referred to our clinic for evaluation and a treatment plan for a pre-prosthetics solution (Figure 3). Accurate impressions of the arches were taken and a diagnostic wax-up was performed in order to better understand the prosthetic needs. A sub-periosteal implant was designed virtually, based on the prosthetics needs. The customized implant was produced with holes for the fixing screws and the integral abutments for the support of the cemented fixed prosthetic rehabilitation.

### 5.3. AM for PSI Manufacturing for Delayed Correction of Post-Traumatic Defects

**Figure 4:**
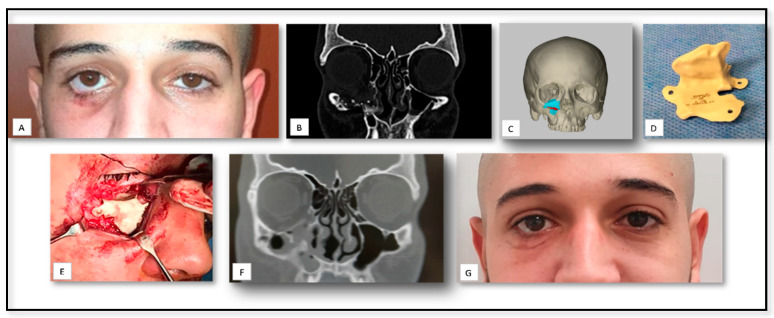
(**A**) A clinical view shows significant right-sided enophthalmos, cicatricial ectropion. (**B**) A preoperative coronal computed tomography (CT) scan shows the defect of the right infraorbital rim. (**C**) Pre-operative 3D planning. (**D**) Individual custom reconstruction implant. (**E**) A post-operative coronal CT scan shows the position of the right infraorbital rim. (**F**) Postoperative implant position. (**G**) Clinical view shows an accepted postoperative esthetic result.

Orbital fractures is a commonly occurring facial bone fractures and clinically important, as they may cause serious complications such as diplopia, extraocular movement limitation, and enophthalmos., resulting in loss of an aesthetically pleasing appearance [59]. A 19-year-old male was referred to our medical center for evaluation and surgical management of injuries sustained 8 month prior, secondary to a gunshot wound injury (GSW) to the right face (Figure 4). Based on his medical history, the first management of his injury included closure of soft tissue on a significant right infra-orbital laceration. Upon initial presentation at our clinic, a clinical examination revealed, facial asymmetry, significant right-sided enophthalmos, cicatricial ectropion and a sensation distribution at the infra-orbital region. Based on a CT scan, a significant avulsed bony injury of his right infraorbital rim and orbital floor was observed. Since the left orbit was not affected, a virtual 3D prototype was designed based on anatomy mirroring of the left orbit. A 3D custom-made implant was created and used to reconstruct the orbital rim and orbital floor. The final result shows a restoration of facial form and contour, with good symmetry and correction of enophthalmos.

### 5.4. AM for Temporomandibular Joint (TMJ) Reconstruction Surgery Due to Oncologic Rresection

**Figure 5:**
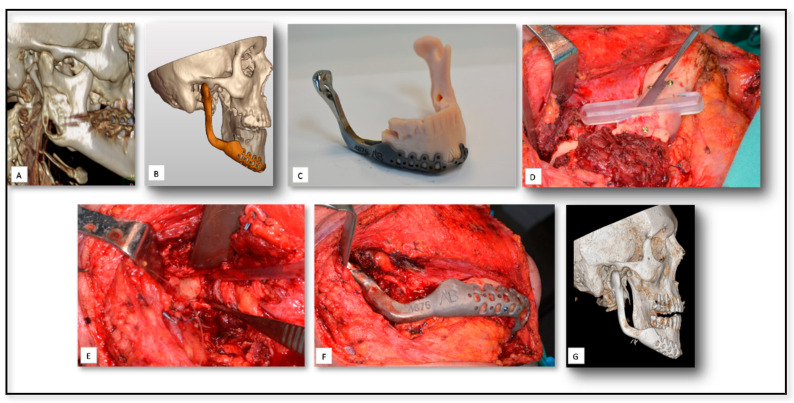
(**A**) A pre-operative 3D CT show the position and dimensions of the metastatic lesion. (**B**) 3D Planning including virtual removal of tumor and the virtual construction of the right temporomandibular joint (TMJ). (**C**) 3D printed stereolitic model and metal implant after virtual removal of Tumor in the right mandibular ramus. (**D**) The use of the cutting guide for accurate resection based on virtual cutting plan. (**E**) Removal of the tumor. (**F**): the placement of the printed TMJ implant. (**G**) Postoperative CT shows the accurate position of the TMJ implant.

Metastatic lesions to the mandible and oral cavity are rare, compromising less than 1% of all malignancy [60]. Here we show a 64-year-old patient with a history of lung signet cell carcinoma that was resected two months prior to his presentation at our clinic (Figure 5). The patient was referred to our institute because of an intra-bony lesion that was noted, radiologically, in his right mandibular ramus. Incisional biopsy was taken from the lesion and metastases from the primary tumor was confirmed, histologically. Resection of the metastasis was planned after discussion with his oncologist. Resection of the tumor required removal of the condyle, resulting in loss of the TMJ but with no articular disc involvement. In this case, a 3D SLA template surgical guide was prepared and used for accurate margins of tumor resection. After resection, a custom metal implant was placed using specific screws. Excellent functional and esthetical results were noted during his follow-up.

### 5.5. AM for Producing PSI for Reconstruction of Large Mandibular Defect after Tumor Resection

**Figure 6:**
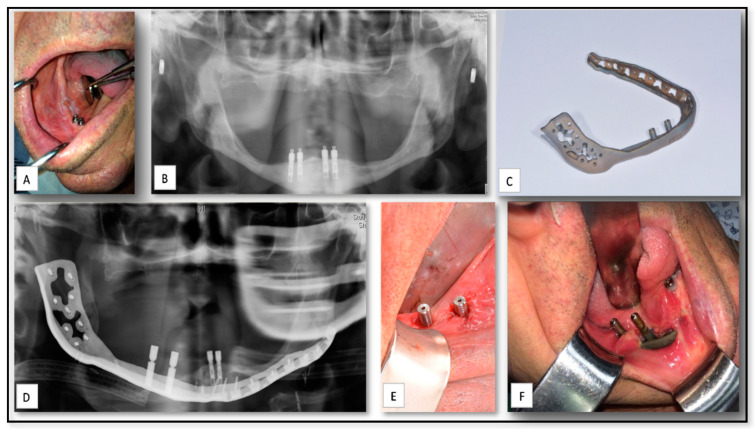
(**A**) A clinical view shows the squamous cell carcinoma (SCC) lesion on at the right posterior mandible. (**B)** A pre-operative panoramic view of the mandible. (**C**) The 3D reconstruction implant of the mandible. (**D**) Post-operative panoramic view shows the implanted reconstruction plate. (**E**) one week after the placement of the reconstruction plate shows the trans-mucosal components of the plate. (**F**) clinical view shows the development of postoperative infection with soft tissue dehiscence.

Squamous cell carcinoma (SCC) of oral cavity is a fatal disease caused by complex interactions between environmental, genomic and epigenetic alterations [61]. Surgical resection with microscopically clear margins of the primary tumor and prophylactic or therapeutic clearance of the neck lymph nodes, followed by various reconstructive approaches, remains the fundamental treatment for Oral Squamous Cell Carcinoma (OSCC) with adjuvant therapy reserved for high-risk disease [62,63,64,65,66]. Here, we present an example of use of 3D approach for reconstruction large mandibular defect following resection of OSCC in the right mandibular body and angle. An 80-year-old man was referred to our institute for evaluation and a treatment plan due to lesion at his right mandible (Figure 6). An incisional biopsy from the lesion confirmed a diagnosis of SCC of the right mandible. A resection of the primary tumor with clear margins was performed and a reconstruction using patient specific plate was placed. The 3D reconstruction plate was planned to include two trans-mucosal implants for subsequent dental rehabilitation. However, this implant failed, and acute infection developed in conjunction with oral and skin fistula. This patient was re-treated, successfully, with free flap fibula reconstruction.

## 6. Current Challenges and Future Directions

Reconstruction of the oral and maxillofacial region is a challenging procedure since it contains several delicate parts (such as maxilla, orbits and the nasal area etc.) with extreme importance in terms of esthetic and functional ability of the patients. Accurate reconstruction surgeries along with minimization of the operation time is of crucial importance to surgeons for improving treatment outcomes.

Nowadays, more than 50% of the clinical trials of 3D printed medical devices are related to the oral and maxillofacial surgery field and most often concern anatomical models for preoperative planning and guides for aiding surgery [67,68]. In the recent years, the 3D printing technology had undergone many adjustments, improvements, enabling an accurate and durable patient-specific model’s creation for complex individualized construct with high fitting properties. These changes lead to the printing of a custom-made patient reconstruction implant where the field of oral and maxillofacial surgery is leading the way in using such devices for clinical use. Various studies have showed the utility of using AM processes as an effective solution for both fabricating PSIs that fit precisely the specific anatomical defects and for pre-operative surgical simulation and planning. As seen also among our clinical examples, the AM technology is applied for printing non-biological components that are used as PSI, intra-operative surgical guides and for pre-operative planning. Indeed, these applications are to be the main indications for using the AM technology in the OMF field.

AM processes are growing and have positively influenced the medical sector by producing biological and non-biological components [69,70]. Recently, humans and animal studies showed some promising results in using bio-printing technology and opened a new avenue for alternative and innovative therapeutic methods for craniofacial defects [71]. Briefly, the bio-printing technology is defined as a single approach combining a set of techniques incorporating cells, biologically active compounds (e.g., growth factors and extracellular matrix components) within or onto a printed substrate. Different material delivery methods and technologies have since been used, including contact bio-printing (e.g., dip pen lithography, [micro]extrusion, and soft lithography) [72,73]; contactless bio-printing (e.g., laser-based forward transfer) [74] and inkjet deposition [75] and other methods. Despite different limitations and obstacles of bio-printing technology (mainly related to scaffold material and scaffold survival), the fabrication of 3D printed scaffolds seem to be a promising alternative approach for bone tissue repair in craniofacial defects [71]. Moreover, the authors believe that once the bio-printing approach is applied successfully for bone tissue repair it can then be extended for soft tissue regeneration and will change, totally, the current management of reconstructive medicine in general, and maxillofacial surgery in particular.

In terms of accurate reconstructive surgery; the accuracy of AM products is still considered to be the main challenge when such objects are printed, knowing that surfaces in contact with a bone at the surgical site need to fit closely to ensure new bone growth and such inaccuracy of printed guides and plates may lead to critical complications. Based on our experience, some printed components are not completely accurate and further minimal adjustment should be performed, intra-operatively, to fit the accurate patient anatomy. This was the intra-operative complication that we needed to deal with. Indeed, most systems used to fabricate biomedical models provide satisfactory accuracy. However, one should take into consideration that the shape, dimensions and anatomic details of prototypes may be affected by errors at any stage of the process, such as CT image acquisition, image manipulation with CAD software, or fabrication and finishing [22,76,77]. Therefore, some parameters should be carefully analyzed to ensure accuracy including: slice thickness when the CAD model is re-sliced, diameter and angle of the laser beam, properties of the used powder particles, and direction of fabrication [77,78]. The authors argue that, to overcome this limitation, work is still needed towards increasing higher-resolution printing, but without sacrificing the strength, handling properties and shape of the final implant.

The non-technology related challenges should not be underestimated, for instance, with one of the limitations being the type of the material to be used. There are very few sets of material available for printing, which present a major setback. Most of the materials used are thermoplastic, while other companies use metal, glass, carbon fibers materials. For instance, when 3D printing bone tissue using SLA only photopolymers could be used, since binder fitting for materials are not suitable in the sintering process.

Staff education is also a main challenge, and developed skills are needed in the manual stages in producing the 3D printed model, and thus staff education is also a major concern in 3D printing process. Most of the PSI products in the present clinical use, as well as in our case series, are produced by titanium material using the SLS technique. For many years, metallic implants have been the most preferred alloplastic material in PSI manufacturing due to their favorable mechanical strength and excellent friction-resistance [79,80,81]. However, different limitations of metal materials are reported including hypersensitivity reactions, osteolysis initiation, MRI incompatibility and the mismatching between the elastic modulus of the metal products and that of normal human bone tissues which may lead to a stress-shielding effect and prosthetic loosening. To overcome this array of limitations and others, a new alloplastic material, PEEK, has emerged and have been considered as promising material for the PSI manufacturing. Briefly, PEEK is a semicrystalline linear polycyclic aromatic thermoplastic belonging to a family of linear aromatic polymers containing ether and ketone linkages [44]. PEEK was first developed in 1978 [79] and has since been used in a wide range of applications owing to its excellent combination of high-temperature performance, chemical resistance, fatigue resistance, lightweight, high yield strength, stiffness, and durability [44]. Various studies conducted with PEEK in reconstruction of complex maxillofacial defects and calvarial defects have shown excellent postoperative esthetic and functional results without any complications [80,81,82]. Although a small number of PSI were performed in the our department using the PEEK material, the authors believe that this material may be very useful for reconstruction of OMF defects, especially, at the non-sensitive sites that do not tolerate a directly applied pressure/load.

In regard of the post-operative complications; one case out of the 16 cases treated at our department showed postoperative loss of the PSI. In this case, extensive edema developed combined with exposure of the PSI and acute infection development. Infection development is well documented in the literature when using PSI devices. In such cases, the results may be catastrophic and may lead ultimately to free flap use in the best scenario. The authors speculate that this complication may be developed due to different causes including patient susceptibility, infection of the surgical wound itself that lead to the PSI exposure, the loss of sufficient soft tissue coverage due to large oncologic resection, stress shielding that leads to loose hardware, and the surface texture of the implant itself. In addition, it should be mentioned that this PSI was implanted with trans-mucosal components in an area with poor keratinized tissue, which may lead to bacterial invasion around these components. Apart from the surgical skills themselves, different studies have aimed to assess the best surface texture modification, mechanically and chemically, for improving the osteointegration process of PSI and showed that surface modification, at the microscale and nanoscale, may support osteoblastic differentiation of normal human osteoblasts and enhance the osteointegration process [83]. In another case, postoperative improper contours of the nasal bridge were noted; this patient was lost subsequently to follow-up. We believe that this may have been due to the soft tissue scars that existed in the surgical site prior to the reconstruction surgery.

A major limitation of the additive manufacturing technologies in general is the fact that there is no consensus practice, nor standardized manufacturing guidelines. Therefore, the same 3D CAD file can be translated into a wide range of models while using different additive methods. All the additive techniques require calibration, processing, and formatting to achieve the required result. However, variability between the machines and the building process may produce a lack of appropriate strength and quality and, thus, a standardized quality measurements are required to ensure that parts built meet appropriate strength and reliability requirements.

Another limitation is supporting the components structures during the printing process. In AM manufacturing, components gain strength through the building process, thus a special concern must be given to the specific processing techniques to support the components structure, and to stand the material weight, external and internal forces from the printing process. Also, additive manufacturing techniques allow internal features to be built but, nonetheless, the geometrical shape and position must be verified [84].

In summary, the utilization of additive manufacturing in craniofacial surgery has significant promise and can extend way beyond the production of custom-fit implants used for large defects in the craniofacial complex resulting from trauma, oncologic surgery, congenital disease, as well as, for surgical stimulation, training and student/resident education. We hypothesize that these enormous potential applications may continue to grow with advancements in imaging, manufacturing, and the widespread availability of more sophisticated printers. In conclusion, from the clinical point of view, additive manufacturing provides a powerful method for fabricating 3D devices based on the CT of individual patients, enabling optimal results in suitable cases. However, more clinical trials with hundreds of cases are needed to build a clear optimal algorithm for the use of this approach.

## Figures and Tables

**Table 1:** List of the 16 cases that were treated at our department between 2015–2020 using the 3D technology including demographic data, surgical discipline, site of surgery, the printed objects, material of choice, intra-operative and post-operative complications. IO: intra-operative, PO: post-operative, TMJ: temporomandibular joint. PEEK: polyetheretherketone, PSI: patient-specific implant, SLA: stereolithographic.

Case Nu.	Age	Sex	Surgical Disciplines	Site	Printed Object	Material	IO. Complication	PO. Complication
1	33	F	Trauma	Mandible	SLA model for pre-bending of reconstruction plate	Resin	−	Mild edema
2	80	M	Oncology	Mandible	PSI for of mandibular body reconstruction including dental implants	Titanium	−	Severe edema, exposure of implant and infection
3	40	M	Trauma	Orbit	PSI for floor of orbit reconstruction	Titanium	−	Mild edema
4	64	M	Oncology	Mandible	PSI of mandibular body with ramus and condyle	Titanium	−	Moderate edema
5	21	M	Trauma	Nose	PSI for nasal bone reconstruction	PEEK	−	Edema and improper contour
6	50	M	TMJ Ankylosis	TMJ	PSI for ramus and condyle reconstruction	Titanium	−	Mild edema
7	49	M	Trauma	Orbit	PSI for floor of orbit reconstruction	Titanium	−	Mild peri-orbital edema
8	20	F	Trauma	Orbital cavity, frontal bone and temporal bone	PSI for temporal and frontal bone reconstruction	PEEK	Loss of accurate fitting	Moderate peri-orbital edema
9	19	M	Trauma	Orbit	PSI for floor of orbit reconstruction	PEEK	−	Mild edema
10	44	F	Trauma	Orbit	PSI for floor of orbit reconstruction	Titanium	Loss of accurate fitting	Mild edema
11	22	F	Facial deformity	Mandible	Surgical cutting guide of mandibular lower border	Resin	Loss of accurate fitting	
12	44	F	Pre-prosthetics	Mandible	PSI of sub-periostal dental implant	Titanium	−	Mild edema
13	50	F	Pre-prosthetics	Maxilla	Surgical guide stent	Titanium	−	
14	71	M	Pre-prosthetics	Maxilla	Surgical guide stent	Titanium	−	
15	66	F	Pre-prosthetics	Maxilla	Surgical guide stent	Titanium	−	
16	56	F	Pre-prosthetics	Maxilla	Surgical guide stent	Titanium	−	
